# Studying Movement-Related Behavioral Maintenance and Adoption in Real Time: Protocol for an Intensive Ecological Momentary Assessment Study Among Older Adults

**DOI:** 10.2196/47320

**Published:** 2023-07-28

**Authors:** Jaclyn P Maher, Derek J Hevel, Kelsey M Bittel, Brynn L Hudgins, Jeffery D Labban, Laurie Kennedy-Malone

**Affiliations:** 1 Department of Kinesiology University of North Carolina Greensboro Greensboro, NC United States; 2 Henry M Goldman School of Dental Medicine Boston University Boston, MA United States; 3 School of Health and Human Sciences University of North Carolina Greensboro Greensboro, NC United States; 4 School of Nursing University of North Carolina Greensboro Greensboro, NC United States

**Keywords:** physical activity, sedentary behavior, aging, multiburst design, ambulatory assessment, experience sampling method, accelerometers, stage of change, dual process, motivation, mobile phone

## Abstract

**Background:**

Older adults struggle to maintain newly initiated levels of physical activity (PA) or sedentary behavior (SB) and often regress to baseline levels over time. This is partly because health behavior theories that inform interventions rarely address how the changing contexts of daily life influence the processes regulating PA and SB or how those processes differ across the behavior change continuum. Few studies have focused on motivational processes that regulate the dynamic nature of PA and SB adoption and maintenance on microtimescales (ie, across minutes, hours, or days).

**Objective:**

The overarching goal of Project Studying Maintenance and Adoption in Real Time (SMART) is to determine the motivational processes that regulate behavioral adoption versus maintenance over microtimescales, using a *dual process framework* combined with ecological momentary assessment and sensor-based monitoring of behavior. This paper describes the recruitment, enrollment, data collection, and analytics protocols for Project SMART.

**Methods:**

In Project SMART, older adults engaging in at least 30 minutes of moderate-to-vigorous intensity PA per week complete 3 data collection periods over 1 year, with each data collection period lasting 14 days. Across each data collection period, participants wear an ActiGraph GT3X accelerometer (ActiGraph, LLC) on their nondominant waist and an ActivPAL micro4 accelerometer (PAL Technologies, Ltd) on their anterior thigh to measure PA and SB, respectively. Ecological momentary assessment questionnaires are randomly delivered via smartphone 10 times per day on 4 selected days in each data collection period and assess reflective processes (eg, evaluating one’s efficacy and exerting self-control) and reactive processes (eg, contextual cues) within the dual process framework. At the beginning and end of each data collection period, participants complete a computer-based questionnaire to learn more about their typical motivation for PA and SB, physical and mental health, and life events over the course of the study.

**Results:**

Recruitment and enrollment began in January 2021; enrollment in the first data collection period was completed by February 2022; and all participants completed their second and third data collection by July 2022 and December 2022, respectively. Data were collected from 202 older adults during the first data collection period, with approximate retention rates of 90.1% (n=182) during the second data collection period and 88.1% (n=178) during the third data collection period. Multilevel models and mixed-effects location scale modeling will be used to evaluate the study aims.

**Conclusions:**

Project SMART seeks to predict and model the adoption and maintenance of optimal levels of PA and SB among older adults. In turn, this will inform the future delivery of personalized intervention content under conditions where the content will be most effective to promote sustained behavior change among older adults.

**International Registered Report Identifier (IRRID):**

DERR1-10.2196/47320

## Introduction

### Background

By 2035, the number of older adults is expected to outnumber children for the first time in the United States [1]. As our society ages, strategies are needed to promote the health and well-being among older adults. Physical activity (PA) offers many physical and mental health benefits [2], yet adults aged ≥60 years are the least active and most sedentary segment of the population [3,4]. Accelerometer-derived data suggest that the average older adult engages in no more than 10 minutes of moderate-to-vigorous intensity PA per day [5]. In addition, most older adults spend 60% to 85% of their waking hours (approximately 9.4 hours each day) engaged in sedentary behavior (SB) [6]. Although short-term interventions have increased PA or decreased SB, individuals often struggle to sustain new behavior patterns in the long term [7,8]. The fact that interventions often fail to have enduring effects on long-term behavior change represents a substantial challenge to health promotion in older adulthood.

### Limitations of Contemporary Theoretical Frameworks

The lack of sustained intervention effectiveness is partly because health behavior theories that inform interventions rarely address how the changing contexts of daily life influence the processes regulating PA and SB or how those processes differ across the behavioral sequence from adoption to maintenance [9]. These are necessary considerations because (1) PA and SB occur within and across days, with optimal levels of these behaviors ideally maintained across the life span, and (2) these behaviors are partly driven by temporal and situational cues such as location, social context, and affective states that rapidly change over time [10]. Moreover, there is a growing body of research that suggests that different pathways guide behavior adoption and maintenance [11-13]. Thus, there is a critical gap in understanding the factors that drive adoption versus maintenance within the context of PA and SB as dynamic, time-varying behaviors. Understanding microtemporal factors (ie, factors that unfold over minutes, hours, and days) within everyday life and how the temporal dynamics of motivational processes contribute to health behavior adoption and maintenance is essential for developing effective behavioral interventions [14].

### Dual Process Models

Dual process models represent a viable framework for investigating microtemporal processes regulating behavioral adoption and maintenance. These models categorize behavioral determinants as either *reflective processes* (intentional, deliberative, slow, and effortful) or *reactive processes* (nonconscious, automatic, fast, and effortless) [15-17]. Reflective processes encompass motivational factors central to many contemporary health behavior theories (eg, attitudes toward a behavior, confidence to perform a behavior, and self-monitoring of goals) [18]. Reflective processes often rely on self-regulation (eg, regulating emotions, coping with stress, managing demands, or exerting self-control) to override a dominant, unwanted behavioral response and translate intentions into goal-directed behavior [19]. Reactive processes represent automatic associations spontaneously activated by stimuli within one’s environment (eg, physical location, surrounding people, and affective state) [20,21].

To adopt a new behavior, a person would likely need controlled effort; willpower and ultimately, a conscious decision to replace the habitual, undesired behavior in a given context. The deliberative overriding of cues and the undesired habitual response would require considerable self-control resources and self-regulatory skills [22,23]. Therefore, strong reflective processes are needed when adopting a new behavior [24-26]. However, once a behavior is well learned and automatic, reactive processes likely dominate the motivations underlying the behavior [27,28], thus promoting behavioral efficiency and making long-term continued behavioral engagement more likely [29,30]. Strong reactive processes are likely needed to maintain engagement in a behavior [11,12].

### Studying Maintenance and Adoption in Real Time

To address existing knowledge gaps, Project Studying Maintenance and Adoption in Real Time (SMART) was designed to test a dual process model mapping the microtemporal mechanisms regulating PA and SB along the continuum from adoption to maintenance in older adults. To accomplish the overall objective, ecological momentary assessment (EMA) methodology and sensor-based monitoring of behavior will be used to intensively capture motivation and behavior over microtimescales and to examine differences in the microtemporal processes underlying the adoption and maintenance of older adults’ PA and SB. Specific research questions to be addressed in this study include the following:

1. To what extent do momentary reflective and reactive motivational processes differentially predict subsequent PA and SB among behavioral adopters and maintainers?
2. To what extent do subject-level patterns in reflective and reactive motivational processes predict behavioral adoption versus maintenance at each wave and across the entire year?
3. How do reflective and reactive motivational processes and behavioral patterns predict change in adopter or maintainer status from wave to wave?

The purpose of this paper is to present the protocol for Project SMART.

## Methods

### Project Overview

**Figure 1:**
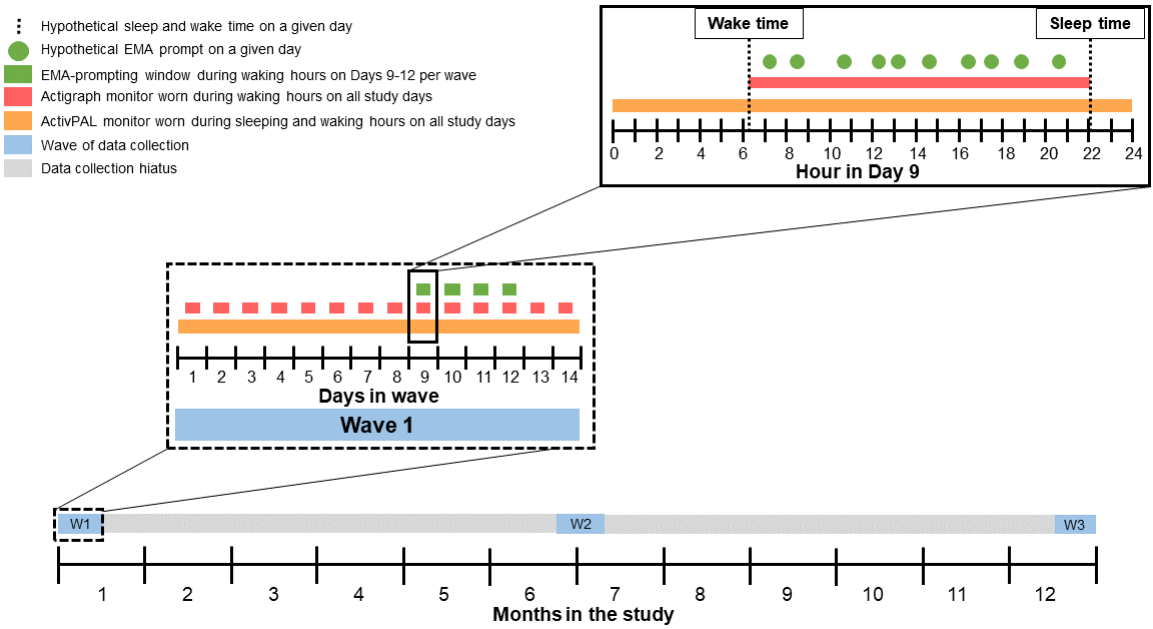
Overview of the study design. EMA: ecological momentary assessment.

Project SMART is a measurement burst design of physically active older adults living in Guilford County, North Carolina. Participants complete three 14-day data collection periods spaced out over the course of 1 year. For the duration of each data collection period, waist-worn and thigh-worn accelerometers measure participants’ PA and SB, respectively. In addition, on days 9 to 12 of each data collection period, participants complete an EMA protocol in which they use a loaned smartphone to answer 10 brief questionnaires per day assessing current behavior, context, feeling states, demands, and motivation to engage in PA and limit SB over the next hour. Figure 1 provides an overview of the study’s design and procedures. Project SMART began enrolling participants in January 2021.

### Target Population and Eligibility

We aimed to enroll 200 older adults who are aged >60 years; living in Guilford County, North Carolina; and currently engage in ≥30 minutes of moderate-to-vigorous intensity PA. Within that sample, we aimed to have approximately 50% (100/200) of older adults classified as PA adopters and 50% (100/200) of older adults classified as PA maintainers through initial and back-end classification (details about the classification are provided in the following sections). We chose minimum levels of PA as the inclusion criteria for behavioral adopters and maintainers rather than levels of SB, because of the relatively high volume and normal distribution of SB in older adults [3,6]. By prioritizing PA adopters and maintainers, we can ensure adequate group numbers, given the frequent low levels of PA among older adults. Adopter or maintainer status classification is repeated at each wave. Exclusion criteria for all participants are the following: (1) unable to speak or read English fluently, (2) functional limitations that prevent older adults from standing or walking on their own for at least 10 consecutive minutes, (3) unable to use a smartphone’s basic functions, (4) unable to hear the auditory signal on the study smartphone, (5) planning to move outside of Guilford County within the next year, or (6) a score indicating impaired cognitive status on a consolidated mental status screener [31].

### Sample Size, Recruitment, and Retention

Sample estimation was performed using Monte Carlo simulations [32], with the expected parameter values derived from aim 1 and aim 2 models fit using our preliminary data (39 participants; 10 observations/day; 4 days). Although missing data in EMA studies of older adults are typically low (ie, almost 90% of EMA studies in older adults achieve compliance of ≥80%) and our preliminary data showed a 96% EMA compliance rate, we conducted simulations using varying patterns of missingness (0%-30%) at varying likelihoods (5%-80%). If missingness occurs at the highest anticipated limits—70 participants per behavioral status group, providing 5 to 7 observations per day over 4 days—we would still obtain 20 to 28 observations per person at each wave. For the study, α was set at .05, and minimum acceptable power was set to 0.80. Given a maximum sample size of 200 participants per model (with simulation sample sizes ranging from 140-200 participants), we achieved power >0.90 for each outcome. To account for possible attrition of up to 30% in both the PA adopter and maintainer groups, we aimed to recruit 100 PA adopters and 100 PA maintainers.

Recruitment efforts targeted fitness centers and older adult programming available within Guildford County, North Carolina. A recruitment method was the electronic distribution of a brief study description with links to videos posted on YouTube that further explained the study, showed the necessary study equipment, and addressed frequently asked questions through cooperating organizations’ listserves. The second major recruitment method was making in-person announcements at exercise classes geared toward individuals aged ≥60 years. In addition, recruitment efforts were conducted through local media coverage of the study and word of mouth. Depending on the recruitment method, participants’ contact information was collected (eg, in-person announcements) or study contact information was provided to individuals.

Owing to the longitudinal nature of this study, retention plans were developed, including (1) obtaining phone numbers, email addresses, and home and mailing addresses of participants and up to 3 other individuals (in case we are unable to reach the participant); (2) scheduling participants for their next data collection period during the current data collection period and providing an appointment reminder card or email after scheduling; (3) sending birthday postcards to participants; and (4) sending study newsletters at 4-month intervals, which are customized to each participant to remind them about their next scheduled data collection period.

### Procedures

Interested individuals complete a telephone screening with a research staff member to determine eligibility. Eligible individuals receive additional details about the study and are able to ask any questions to a research staff member. Participants are then scheduled to attend an introductory session. A week before an introductory session, participants receive a link to a questionnaire via email to provide electronic consent for the study and complete a baseline assessment of their motivation for PA and SB, self-reported levels of PA and SB, and demographic and contact information. Participants are asked to complete the baseline assessment before their introductory session.

An introductory session is conducted on day 1. Introductory sessions are conducted every 3 weeks, and as a result, participants are scheduled on a rolling basis. A research staff member meets with individual participants (or participants from the same household) in person on campus, at a community location, or via Zoom (Zoom Video Communications), depending on the participant’s preference. Participants meeting via Zoom receive study equipment in person at a community location, or study equipment is delivered to their home. During the introductory session, the research staff member reviews the consent document with the participant and answers any questions. Then, participants are familiarized with the study procedures and trained on 2 accelerometers to be worn for the next 14 days. Accelerometers include an ActiGraph GT3X-BT accelerometer to be worn on their nondominant waist during all waking hours (except when swimming or bathing) to provide a device-based measure of PA and an ActivPAL3 micromonitor to be worn on their thigh during all sleeping and waking hours to provide a device-based measure of SB. Participants begin wearing the activity monitors at the introductory session. Participants are given a sleep and wake time log and an activity monitor log to complete over the 14-day study period. On the sleep and wake time log, participants indicate for each day the time they got out of bed to start their day (ie, wake time) and the time they began trying to fall asleep at the end of the day (ie, sleep time). On the activity monitor log, participants report any waking times during which they were not wearing their activity monitor and a brief explanation of the reason. These logs will be used to validate activity monitor nonwear algorithms.

A week later, on day 8, a research staff member meets the participant or participants again (in person or via Zoom) to train them on how to use a smartphone preloaded with a commercially available, app, MovisensXS, on which they answer brief EMA questionnaires. To ensure that the MovisensXS app is functioning as intended, all participants are loaned a Motorola Moto G Power mobile phone, a compatible device for the MovisensXS app. Participants receive a practice questionnaire at 7:30 PM that same evening to practice accessing the questionnaire and familiarizing themselves with the questions and response formats. The practice questionnaire will not be included in the analytic data set.

On days 9 to 12, participants receive prompts to complete the EMA questionnaire 10 times per day as part of the study procedures. Participants select 1 of 3 EMA prompting schedules for the data collection period based on their self-reported usual wake time. Available EMA prompting schedules deliver questionnaires between 7 AM and 9 PM, between 8 AM and 10 PM, or between 9 AM and 11 PM. Across all prompting schedules, the day is divided into intervals of 1 hour and 25 minutes. Participants receive 1 EMA prompt randomly during each interval. There is an imposed minimum 30-minute buffer period between the random EMA prompts. Participants are alerted to the EMA prompt via an auditory signal. Participants can adjust the mobile phone volume but are encouraged, when able, to have the mobile phone volume turned up to maximum. Upon hearing a prompt, participants are asked to complete a brief EMA questionnaire on the mobile phone. If no entry is made, the app emits up to 3 reminder signals at 5-minute intervals. If a signal occurs during an incompatible activity (eg, driving or sleeping), participants are instructed to ignore it or complete that activity before answering. Each EMA questionnaire contains 20 items and is expected to take 2 to 3 minutes to complete (refer to the *EMA* *Measures* section for details). Upon going to bed on day 12, participants will have completed the EMA protocol but continue to wear their activity monitors until waking on day 15.

On day 15, participants return their equipment at an in-person appointment or mail the equipment back to the research team using a prepaid United Parcel Service envelope provided to them. Participants also complete a post–data collection period questionnaire, which assesses life events, affective experiences during movement behaviors, behavior over the data collection period, and feedback about the data collection period.

Study procedures are identical for each data collection period, except that, at the second and third data collection periods, participants are given the option to complete a 1-hour, condensed training session reviewing both the activity monitors and smartphone on day 1 as opposed to 2 separate 1-hour training sessions spaced through the data collection period.

For their participation in the study, participants can earn up to US $100 for each data collection period. Participants are compensated with US $60 for completing the data collection period. If participants answer at least 80% of the questionnaires on the smartphone, they receive US $40 as bonus. If participants complete all 3 data collection periods, they receive US $40 as bonus at the third data collection period. Therefore, participants can earn up to US $340 for this study.

### Measures

#### Adopter and Maintainer Status

##### Overview

To determine adopter and maintainer status, we use a staged classification model using self-reported behavior initially, with subsequent selective verification via accelerometer. Overall, 2 weeks of accelerometer data will be collected to more reliably differentiate adopters and maintainers, given that self-report measures are subject to recall biases and social desirability [33] and having only 1 week of accelerometer data may not provide an accurate representation of participants’ typical levels of PA and SB.

##### PA Adopter and Maintainer Status

Initial eligibility based on PA level is determined during the telephone screening before study enrollment using items assessing moderate-to-vigorous intensity PA from the International Physical Activity Questionnaire Short Form [34]. Items include the number of days in the past 7 days in which participants engaged in moderate-to-vigorous intensity PA and the typical amount of time spent engaged in that activity on one of those days. Individuals engaging in <30 minutes of moderate-to-vigorous intensity PA over the past 7 days are excluded. Here, 30 minutes was chosen as the minimum level of PA to increase the likelihood of capturing moments of PA during the wave (to minimize the extent to which we have a skewed distribution for data analysis where PA serves as the outcome).

**Figure 2:**
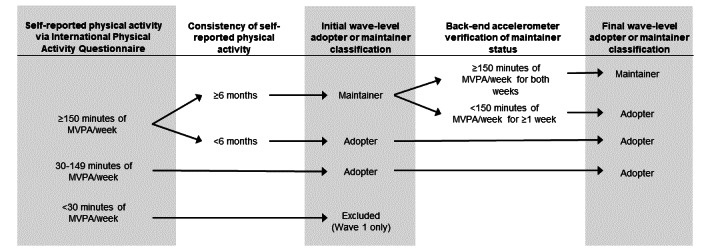
Overview of adopter or maintainer classification procedures at each data collection period. MVPA: moderate-to-vigorous intensity physical activity.

Then, during the administration of the baseline electronic questionnaire, participants will complete the Physical Activity Stage of Change Questionnaire [35], where they indicate whether they are currently engaging in 150 minutes of PA per week and, if so, how long they have been engaging in that level of PA. Individuals indicating <150 minutes of moderate-to-vigorous intensity PA per week will be classified as adopters. Those indicting that they engage in ≥150 minutes of moderate-to-vigorous intensity PA but for <6 months will also be classified as adopters. Those indicating ≥6 months of that PA level will be categorized as maintainers. As overestimation of PA is a common methodological weakness associated with self-reported, retrospective measures of PA [33], PA maintainer status will be confirmed via accelerometry. On the back end of each wave of data collection, maintainer status will be confirmed by examining ActiGraph-derived minutes of moderate-to-vigorous intensity PA during the wave. Participants who meet the 2018 US Federal PA guidelines according to ActiGraph-derived data during both weeks of data collection will remain as maintainers. Those who do not meet 2018 US Federal PA guidelines according to ActiGraph-derived data for one or both weeks are recategorized as adopters. The staged classification model is depicted in Figure 2. This initial and back-end classification will be performed at each data collection period.

##### SB Reduction Adopter and Maintainer Status

Although our inclusion criteria are based on PA adopter and maintainer status, we also determine SB reduction adopter and maintainer status via the baseline questionnaire before each data collection period using items from the International Physical Activity Questionnaire [34], assessing weekday and weekend-day SB. Those indicating sitting for >8 hours per day will be considered as SB reduction adopters. Individuals indicating sitting for ≤8 hours per day for <6 months are also categorized as adopters; those sitting for ≤8 hours per day for ≥6 months are classified as maintainers. We confirm SB reduction adopter or maintainer status using ActivPAL-derived data and follow similar recategorization procedures for PA. We chose sitting for ≤8 hours per day as the cutoff for adopter or maintainer status because older adults who sit for >8 hours per day have high risk for premature death and other chronic conditions [36].

#### EMA Measures

EMA measures real-time experiences of reflective and reactive factors (refer to Multimedia Appendix 1 for details about reflective and reactive EMA items). EMA data are stored on the study-provided mobile phone after each entry and uploaded to a secure server when a participant enters a Wi-Fi–enabled area. If a participant does not have access to Wi-Fi, all responses are stored on the mobile phone until uploaded by a research assistant upon completion of the data collection period. All EMA items are either taken directly or modified from established measures. Construct-general (instead of behavior-specific) reflective EMA items begin with the stem, “Right before the phone went off...” to capture momentary reports. Each construct-general reflective factor is assessed using a single item appearing in every EMA prompt. Construct-general reflective EMA items will assess momentary self-efficacy [37], deliberation [38], self-control [39], demands [40], stress coping [41], and emotion regulation [42]. In addition, we also assess behavior-specific reflective factors through EMA. Behavior-specific reflective factors include single-item assessments of intentions, self-efficacy, and planning to engage in PA and reduce SB over the next hour [43,44]. At each EMA prompt, 2 items assessing behavior-specific reflective factors are randomly delivered to participants. Therefore, each behavior-specific reflective factor will be assessed 3 to 4 times per day. Reactive EMA items will assess current positive and negative affect. Positive and negative affect assessments each use a domain-specific approach assessing 5 affective domains; however, each EMA prompt will only contain 3 affect-related items randomly delivered. The average of the available items for each construct will be used to create momentary positive and negative affect composite scores. Additional reactive factors include current functional stability of habits (1 item), physical context (2 items), and social context (1 item) at each EMA prompt [45,46]. Each EMA prompt is date and time stamped to assess the temporal context. Temporal context data will be used to create dummy-coded variables related to the day of week (ie, weekend day vs weekday) and time of day (eg, morning vs other times of day). Participants also self-report their behavior at every EMA prompt (2 items). Each EMA prompt contains 20 items and takes 2 to 3 minutes to complete.

#### Device-Based PA and SB

The ActiGraph GT3X-BT accelerometer provides a device-based measure of PA. Participants are instructed to wear the ActiGraph device on their nondominant waist during all waking hours. If participants engage in activities where the monitor could get wet (eg, swimming and showering or bathing), they are instructed to remove it. Data are collected in 30-second epochs. The ActiGraph has been used extensively in large epidemiologic studies to assess PA and validated in both laboratory and field settings [3,5]. Existing count-based intensity thresholds inform time spent in specific intensities of PA. The count threshold for moderate-to-vigorous intensity PA (≥2020 counts/min) was designed and validated specifically to assess PA in populations across the adult life span and has been used extensively in epidemiologic studies and intervention trials [3]. PA is operationalized as the total time spent engaged in moderate-to-vigorous intensity PA in the 60 minutes after the EMA prompt. Any occasion where the ActiGraph records 0 minutes of valid wear time in the 60 minutes after the EMA prompt will be considered as nonwear and coded as missing data. Accelerometer recordings will be date and time stamped to link with EMA data.

The ActivPAL3 microaccelerometer provides a device-based measure of SB. Participants will be instructed to wear it on their anterior thigh during all sleeping and waking hours. Participants’ ActivPALs are waterproofed using heat-sealed polymer plastic tubing to ensure that wearing it during water-based activities does not damage the monitor. Owing to the placement of the ActivPAL on the thigh, the monitor is better able to detect different postures (ie, sitting, lying, and standing) compared with waist-worn accelerometers such as the ActiGraph [47]. The ActivPAL has been used in large epidemiologic studies and intervention trials and has undergone validation in both laboratory and field settings [4,48,49]. ActivPAL uses proprietary algorithms to categorize behavior in 15-second epochs as time spent in SB. SB is operationalized as total time spent engaged in SB in the 60 minutes after the EMA prompt. Any occasions where the ActivPAL was not worn for the entire 60 minutes after the EMA prompt will be considered as nonwear and coded as missing data.

### Data Analysis Plan

#### Overview

An overview of key study measures and their role in data analysis is presented in Table 1.

**Table 1:** Overview of key study measures and their role in data analysis.

Construct	Measure	Aim 1	Aim 2		Aim 3
			Hypothesis 2a	Hypothesis 2b	

Reflective factors	EMA^a^ (10 prompts/day)	Predictor	Stage 1—outcome Stage 2—predictor	Stage 1—predictor	Predictor
Reactive factors	EMA (10 prompts/day)	Predictor	Stage 1—outcome Stage 2—predictor	Stage 1—predictor	Predictor
Physical activity	ActiGraph GT3X-BT	Outcome	N/A^b^	Stage 1—outcome Stage 2—predictor	Predictor

Sedentary behavior	ActivPAL3 micro	Outcome	N/A	Stage 1—outcome Stage 2—predictor	Predictor

Adopter or maintainer status	Initial self-report classification—International Physical Activity Questionnaire Back-end accelerometer classification—ActiGraph GT3X-BT and ActivPAL3 micro	Moderator	Stage 2—outcome	Stage 2—outcome	Outcome

^a^EMA: ecological momentary assessment.

^b^N/A: not applicable.

#### Aim-1 Analytic Plan

Hierarchical linear modeling [50] will be used to determine the extent to which momentary reflective and reactive motivational processes are associated with subsequent PA and SB in the 60 minutes after the EMA prompt. Separate, 2-level (observations within participants) random effects models will be estimated for both PA and SB, with only 1 reflective or reactive factor tested at a time, resulting in a total of 18 reflective factor models and 10 reactive factor models. Reflective and reactive variables will be person-mean centered at level 1 and grand-mean centered at level 2 to partition variance in outcomes owing to within-subject changes and between-subject differences, respectively [51]. Maintainer status at baseline will be entered as a person-level covariate; both within-level and cross-level interactions with reflective and reactive variables will be tested. In addition, it is not uncommon for PA data to be positively skewed. Should model assumptions concerning normality be violated, we will log transform dependent variables and refit the model or models or shift to hierarchical generalized linear modeling should a non-Gaussian link function be more appropriate [50]. The fixed effects for interactions between maintainer status and reflective and reactive variables on PA and SB will be of primary interest in investigating this aim. It is because of our specific interest in these interactions that we will not include multiple reflective and reactive factors in the same model. Finally, given the number of model parameters to be estimated across all models, we will use false discovery rate to maintain a type-1 error rate within acceptable levels (0.05) while limiting commensurate type-2 error inflation that occurs with other techniques used to control family-wise error rates (eg, Bonferroni correction) [52].

#### Aim-2 Analytic Plan

Aim 2 is designed to determine the extent to which subject-level patterns in reflective and reactive motivational processes predict behavioral adoption versus maintenance. Analyses for aim 2 will use a novel, 2-stage approach for each hypothesis using the stand-alone program, MixWILD [53]. In stage 1, mixed-effects location scale modeling [54,55] is used to model between-subject and within-subject variability in momentary reflective and reactive motivational processes. With heterogeneous variability, mixed-effects location scale modeling extends the typical hierarchical linear model to allow the incorporation of covariates in the modeling of between-subject and within-subject variability. Thus, we will estimate separate 2-level random effects models, controlling for relevant demographic and temporal covariates (refer to information about covariate screening in the previous section). We will estimate effects of random within-subject means (ie, subject-level average of a given construct based on all available occasions of data—location effect), variance (ie, subject-level degree of intraindividual variability on a given construct across all available occasions of data—scale effect), and slope (ie, subject-level average strength of an association between 2 variables across all available occasions of data). Within-subject mean, variance, and slope random effect estimates generated in stage 1 will be retained for use in the stage-2 analysis.

In stage 2, the within-subject mean, variance, and slope estimates of reflective or reactive variables will serve as predictors of likelihood of categorization as a behavioral adopter or maintainer (coded as 0 or 1, respectively) in single-level logistic regression models [53]. Separate single-level logistic regressions will be tested for each wave and for the entire year, with associated location and scale estimates incorporated as covariates. The dependent variables will be maintainer status at the end of each wave (for single-wave models) and whether participants retained the classification as a maintainer across all 3 waves (year model). In addressing each hypothesis associated with aim 2, model refinement will be informed by the magnitude and significance of individual beta coefficients and the impact on omnibus model performance assessed via change in –2 log likelihood. Final conditional models will be examined for improvement in classification accuracy over null models and for the individual performance of subject-level mean and variance effects in reflective and reactive factors and subject-level slopes regarding associations between reflective and reactive factors and subsequent behavior in predicting maintainer status. Again, we will use false discovery rate in each stage to control type-2 error rates [52].

#### Aim-3 Analytic Plan

Aim 3 is designed to investigate the extent to which reflective and reactive motivational processes and behavioral patterns predict change in adopter or maintainer status from wave to wave. For this exploratory aim, we will apply latent Markov modeling to examine predictors of individuals’ status change over waves (ie, adopter to maintainer or maintainer to adopter) [56]. Specifically, we will estimate adopter or maintainer status using observed status and test whether subject-level mean, variance, and slope random effects generated in aim-2 analysis can be used to predict the likelihood of transitioning from one status category to another. Given the power concerns and the exploratory nature of this aim, we will test each reflective or reactive variable within a separate model.

### Ethics Approval

All study procedures were approved by the University of North Carolina Greensboro institutional review board (20-0216) in January 2020.

## Results

**Figure 3:**
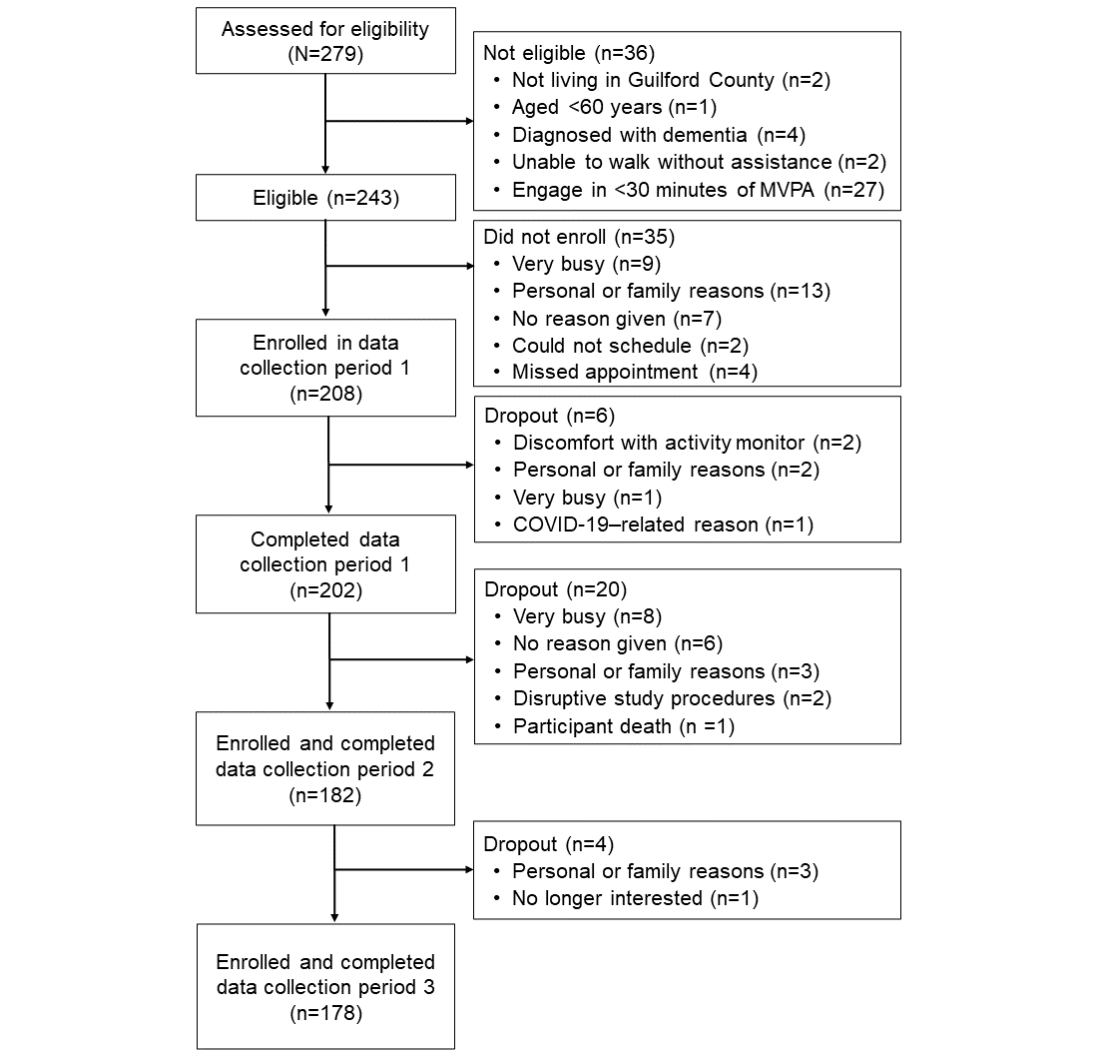
CONSORT (Consolidated Standards of Reporting Trials) flow diagram. MVPA: moderate-to-vigorous intensity physical activity.

Funding for Project SMART was awarded in April 2020. Additional COVID-19 protocols were approved by the University of North Carolina Greensboro’s Office of Research Engagement in December 2020. Participant recruitment and enrollment began in January 2021. As of February 2022, a total of 202 participants enrolled and completed the first data collection period. As of July 2022, overall, 90.1% (182/202) of participants completed their second data collection period. As of December 2022, overall, 88.1% (178/202) of participants completed the third and final data collection period. Among those who dropped out of the study between data collection period 1 and 2 and between 2 and 3 (n=24), reasons for attrition included no longer being interested in the study (n=1, 4%), personal or family reasons (n=6, 25%), being very busy (n=8, 33%), having found the smartphone protocol to be disruptive (n=2, 8%), or no reason was given as they were unable to be contacted (n=6, 25%). A participant (1/24, 4%) also passed away during a scheduled break from data collection between data collection periods 1 and 2. A flow diagram showing participant progress through the recruitment, screening, enrollment, and data collection processes is shown in Figure 3.

Of those who enrolled in and completed the first data collection period and provided relevant information, most identified as female (female: 142/199, 71.4%; male: 57/199, 28.6%) and White (Asian: 1/199, 0.5%; Black or African American: 47/199, 23.6%; White: 148/199, 74.4%; and ≥2 races: 3/199, 1.5%). The average age of the sample was 69.9 (SD 5.7; range 60-85) years. Most participants were overweight (61/194, 31.4%) or obese (49/194, 25.3%; mean BMI 27.1, SD 5.5 kg/m^2^). The median annual income in the sample was US $60,000 to US $79,999. Approximately 22.6% (45/199) of the participants reported <US $40,000, and 21.1% (42/199) reported >US $100,000 as annual income. After back-end classification, approximately two-thirds (127/201, 63.2%) of the sample were classified as PA adopters, and the remaining (74/201, 36.8%) were classified as PA maintainers.

## Discussion

### Summary

Project SMART will determine the motivational processes that regulate behavioral adoption versus maintenance over microtimescales, using a dual process framework combined with EMA and sensor-based monitoring of behavior. Applying a within-subject approach permits a refined examination of motivation-behavior relations as naturally occur in everyday life across the behavior change continuum. This paper describes our approach to understanding these relations.

### Methodological Challenges and Decisions

To enhance the likelihood of capturing moments of PA during data collection periods, this study requires that older adults currently engage in at least 30 minutes of self-reported PA per week. There are many older adults who engage in <30 minutes of self-reported PA per week. An important direction for future studies is to examine the motivational processes at play in inactive and insufficiently active older adults. Such studies would complement the proposed study by identifying microtemporal motivational processes regulating health behaviors among older adults at other key points in the behavior change continuum such as contemplation (ie, intending to change behavior in the next 6 months) or preparation (ie, intending to change behavior in the next 30 days and sporadically engaging in desired health behavior) [57]. Future studies expanding our approach to additional points in the behavior change continuum would enable temporally sensitive and context-sensitive interventions applicable to a wide range of older adults. Minimum levels of PA are used as an inclusion criterion rather than levels of SB because of the relatively high volume and normal distribution of SB in older adults [4,6].

In this study, participants are loaned Motorola Moto G Power mobile phones to complete the EMA protocol. This decision was made for several reasons. First, the MovisensXS app is available only on Android devices. As most smartphone owners in the United States use an iOS device [58], the research team did not want to exclude a large proportion of potential participants owing to the operating system of the personal device. Second, MovisensXS tests specific devices and operating systems to determine their potential for app performance. The Moto G series of devices has been shown to perform well in executing various features of the MovisensXS app. Furthermore, the MovisensXS app currently requires an operating system of Android 4 or higher. Providing a loaned mobile phone ensures that the recommended operating system is in use and that the device is optimized for performance. Finally, evidence suggests that adults have improved compliance when completing an EMA protocol on a loaned mobile phone as opposed to one’s personal device [59]. This may be because participants are more sensitive to novel app-based notifications from a new device as opposed to their personal device on which they may be used to ignoring notifications. In this study, the research team weighed the costs and limitations associated with buying a limited number of devices (n=30) and the benefits associated with optimal app performance. Another important consideration for providing participants with devices is that introducing a novel device may require additional training, depending on the participant population. In this study, because of the need to meet with participants (in person or over Zoom) to train them on wearing the activity monitors, there was a built-in opportunity to train them on the smartphone device as well.

### Strength of Project SMART

Previous studies that aimed at understanding the motivational processes regulating movement-related behaviors in older adults have used retrospective or summary-based questionnaires, which may be prone to recall biases and misremembering, to capture typical levels of behavior [10]. Unlike these traditional (between-person) methods, our approach recognizes that movement behaviors change within individuals and across days and that the pattern of variation in movement behaviors may differ between people. Furthermore, this study addresses those methodological weakness by pairing EMA of older adults’ current motivation, affect, and contexts with device-based movement behavior. This study capitalizes on the fact that mobile phones have become ubiquitous and easy to use in daily life, even among older adults [60]. Previous studies have documented that smartphone-based EMA studies of movement-related behavior are feasible, acceptable, and valid among diverse groups of older adults [61,62]. However, this is the first known movement behavior EMA study to intensively assess older adults 10 times per day. This study will enhance our knowledge about how EMA can be used among older adults in combination with other types of real-time behavioral measures (ie, accelerometers) to capture momentary, within-day effects of reflective and reactive motivational processes on older adults’ PA and SB.

This study will help to generate more definitive conclusions about how reflective and reactive processes operate to influence PA and SB from adoption to maintenance. In addition, this study will not only explore the role of specific levels of constructs (eg, degree of confidence) or the presence of a particular construct (eg, alone vs not alone) but also explore patterns of stability (or instability) in reflective and reactive processes and the influence those patterns have on behavior across the behavior change continuum. This study is a necessary and fundamental step toward refining health behavior theories to predict health behaviors more accurately as they unfold over everyday life [14].

In summary, Project SMART is well suited to address gaps in the current understanding of the motivational processes that regulate movement behaviors along the continuum of adoption to maintenance. By intensively assessing movement behaviors and motivational antecedents, Project SMART aims to generate a great understanding of the within-person, reflective, and reactive motivational processes regulating behavior and thus will provide information to identify intervention targets in the context of everyday life. Specifically, novel intervention approaches such as just-in-time approaches [63] that can respond to participants in real time based on passively collected or participant-reported data may benefit from our more ecologically valid, real-time approach. Ultimately, this study is necessary to develop behavioral interventions that deliver personalized intervention content (eg, motivational messages and prompts to increase PA or reduce SB) under the temporal and contextual contexts when that content will be most effective to promote sustained behavior change among older adults.

## Appendix

Multimedia Appendix 1 Ecological momentary assessment items.

Multimedia Appendix 2 Peer-review reports from Behavioral Medicine, Interventions and Outcomes (BMIO) Study Section Risk, Prevention and Health Behavior Integrated Review Group - Center for Scientific Review (National Institutes of Health, USA).

## Abbreviations

EMA: ecological momentary assessment
PA: physical activity
SB: sedentary behavior
SMART: Studying Maintenance and Adoption in Real Time

## Data Availability

The data sets generated and analyzed during this study are available from the corresponding author upon reasonable request.
