# Supplementary material for: Studying Movement-Related Behavioral Maintenance and Adoption in Real Time: Protocol for an Intensive Ecological Momentary Assessment Study Among Older Adults
Source: JMIR Res Protoc. 2023 Jul 28;12:e47320. doi: 10.2196/47320 (PMC10422169; doi:10.2196/47320)
Supplement: Multimedia Appendix 1 [file resprot_v12i1e47320_app1.docx]

| Variable | Example item | # of items | Response options | Frequency | |  |
| --- | --- | --- | --- | --- | --- | --- |
| **Reflective processes** | | | | | | |
| Self-efficacy | Right before the phone went off, I felt confident that I would be able to achieve most of the goals that I set for myself. | 1 | Slider scale—0 (not at all confident) to 100 (very confident) | Every prompt | |  |
| Deliberation | Right before the phone went off, I was having a hard time making decisions. | 1 | Slider scale—0 (not at all) to 100 (very much so) | Every prompt | |  |
| Self-control | Right before the phone went off, I felt in control. | 1 | Slider scale—0 (not at all) to 100 (very much so) | Every prompt | |  |
| Demands | Right before the phone went off, I was juggling several things at once. | 1 | Slider scale—0 (not at all) to 100 (very much so) | Every prompt | |  |
| Stress coping | Right before the phone went off, I was managing my stress levels. | 1 | Slider scale—0 (not at all) to 100 (very much so) | Every prompt | |  |
| Emotion regulation | Right before the phone went off, I was controlling my emotions. | 1 | Slider scale—0 (not at all) to 100 (very much so) | Every prompt | |  |
| **Behavior-specific reflective processes** | | | | | Across all behavior-specific reflective items, 2 items randomly asked at each prompt | |
| PA Intentions | Over the next hour, I intend to engage in at least 10 minutes of physical activity. | 1 | Slider scale—0 (strongly disagree) to 100 (strongly agree) |  | |  |
| SB reduction intentions | Over the next hour, I intend to limit the time I spend sitting to less than 45 minutes. | 1 | Slider scale—0 (strongly disagree) to 100 (strongly agree) |  | |  |
| PA Self-efficacy | Over the next hour, I feel confident that I can engage in at least 10 minutes of physical activity. | 1 | Slider scale—0 (not at all confident) to 100 (very confident) |  | |  |
| SB reduction self-efficacy | Over the next hour, I feel confident that I can limit the time I spend sitting to less than 45 minutes. | 1 | Slider scale—0 (not at all) to 100 (very much so) |  | |  |
| PA Plans | I have made a detailed plan for how I am going to engage in at least 10 minutes of physical activity over the next hour. | 1 | Slider scale—0 (strongly disagree) to 100 (strongly agree) |  | |  |
| SB reduction plans | I have made a detailed plan for how I am going to limit the time I spend sitting to less than 45 minutes over the next hour. | 1 | Slider scale—0 (strongly disagree) to 100 (strongly agree) |  | |  |
| **Reactive processes** | | | | | | |
| Habit functional stability | Right before the phone went off, I was following my usual routine. | 1 | Slider scale—0 (not at all) to 100 (very much so) | Every prompt | |  |
| Positive affect | Right before the phone went off, how HAPPY/RELAXED/CALM/EXCITED/ENERGETIC were you feeling? | 5 | Slider scale—0 (very bad) to 100 (very good) | 3 items are randomly asked at each prompt | |  |
| Negative affect | Right before the phone went off, how SAD/FRUSTRATED/ANXIOUS/UPSET/TIRED were you feel? | 5 | Slider scale—0 (low) to 100 (high) | 3 items are randomly asked at each prompt | |  |
| Physical context | Where are you just before the phone went off? | 1 | Select 1 option—indoors or outdoors | Every prompt | |  |
| Physical context | Where were you indoors? | 1 | Select 1 option—my home, shared community living space, work, store, restaurant, someone else’s home, fitness facility, community center, church, car or bus, or other | Participant received this item if they responded “Indoors” to the item, “Where were you just before the phone went off?” | |  |
| Physical context | Where were you outdoors? | 1 | Select 1 option—my home, my neighborhood, park or trail, community center, fitness facility, restaurant, shopping center, someone else’s home, sidewalk, parking lot, car/bus, or other | Participant received this item if they responded “Outdoors” to the item, “Where were you just before the phone went off?” | |  |
| Social context | Who are you with (in-person and/or virtual) just before the phone went off? | 1 | Select multiple options—I was alone, spouse or partner, family member(s), friend(s), neighbor(s), coworker(s), other types of acquaintances, pet or animal companion, or people I do not know | Every prompt | |  |
| **Self-reported behavior** | | | | | | |
| Current activity | What were you doing just before the phone went off? | 1 | Select multiple options—watching television/movies, using a computer/tablet/phone, reading, socializing, doing hobbies, physical activity or exercise, running errands, household chores, sleeping or napping, riding in a car, eating or drinking, childcare, self-care activities, or other | Every prompt | |  |
| Currently sitting | Were you sitting while doing that activity? | 1 | Select 1 option—yes or no | Every prompt | |  |

^a^Each ecological momentary assessment prompt will contain 20 items. Ecological momentary assessment prompts are date and time stamped to provide information about the temporal context, a reactive factor.
