# Supplementary material for: Studying Movement-Related Behavioral Maintenance and Adoption in Real Time: Protocol for an Intensive Ecological Momentary Assessment Study Among Older Adults
Source: JMIR Res Protoc. 2023 Jul 28;12:e47320. doi: 10.2196/47320 (PMC10422169; doi:10.2196/47320)
Supplement: Multimedia Appendix 2 [file resprot_v12i1e47320_app2.pdf]

SUMMARY STATEMENT
(Privileged Communication)

PROGRAM CONTACT:
LISBETH NIELSEN
301-402-4156
nielsenli@nia.nih.gov

Release Date: **10/22/2019**
Revised Date:

Application Number: **1 R15 AG066950-01**

Principal Investigator

MAHER, JACLYN PARENTE

Applicant Organization: UNIVERSITY OF NORTH CAROLINA GREENSBORO

Review Group: **BMIO**
Behavioral Medicine, Interventions and Outcomes Study Section

Meeting Date: **10/03/2019**
Council: **JAN 2020**
Requested Start: **04/01/2020**
RFA/PA: **PAR18-714**
PCC: **2BPDILN**

Project Title: **Microtemporal Motivational Processes Regulating Health Behavior Adoption and Maintenance in Older Adults**
SRG Action: **Impact Score:23 Percentile:6 +**
Next Steps: **Visit https://grants.nih.gov/grants/next_steps.htm**
Human Subjects: 30-Human subjects involved - Certified, no SRG concerns
Animal Subjects: 10-No live vertebrate animals involved for competing appl.
Gender: 1A-Both genders, scientifically acceptable
Minority: 1A-Minorities and non-minorities, scientifically acceptable
Age: 8A-Only Older Adults, scientifically acceptable

Project Year	Direct Costs Requested	Estimated Total Cost
1	300,000	423,820
TOTAL	300,000	423,820

ADMINISTRATIVE BUDGET NOTE: The budget shown is the requested budget and has not been adjusted to reflect any recommendations made by reviewers. If an award is planned, the costs will be calculated by Institute grants management staff based on the recommendations outlined below in the COMMITTEE BUDGET RECOMMENDATIONS section.

1R15AG066950-01 Maher, Jaclyn

COMMITTEE BUDGET RECOMMENDATIONS

RESUME AND SUMMARY OF DISCUSSION: This application proposes to study the micro-temporal processes that drive adoption and maintenance of physical activity and sedentary behavior among older adults. The study is likely to result in multiple peer-reviewed publications, will strengthen the research environment, and will expose undergraduate students to a research experience. The investigator is excellent and has an undergraduate research mentoring history. During discussion many strengths were noted: preliminary data demonstrating feasibility and proof of concept; valid assessments of key variables; and carefully described analytic plan. However, some weaknesses were noted including: longitudinal data seems poorly integrated with the outcome metrics; and feasibility of recruitment was questioned. The strengths clearly outweighed any weaknesses and the committee concluded that the potential overall impact was high.

DESCRIPTION (provided by applicant): Older adults struggle to maintain newly initiated levels of physical activity (PA) or sedentary behavior (SB) and often regress back to baseline levels over time. This is in part because health behavior theories that inform interventions rarely address how the changing contexts of daily life influence the processes regulating PA and SB, or how those processes differ across the behavior change continuum from adoption to maintenance. These are necessary considerations as PA and SB occur within and across days with optimal levels of these behaviors ideally maintained across the lifespan and are in part driven by temporal and situational cues that rapidly change over time. To date, little research has focused on motivational processes that regulate the dynamic nature of PA and SB adoption and maintenance on micro timescales (i.e., across minutes, hours, or days). Thus, we propose to determine the motivational processes that regulate behavioral adoption versus maintenance over micro timescales, using a dual-process framework combined with Ecological Momentary Assessment (EMA) and sensor-based monitoring of behavior. Older adults identified as PA adopters (n=100) and PA maintainers (n=100) will complete 3 data collection waves over 1 year, with each wave lasting 14 days. EMA questionnaires will be randomly delivered via smartphone 10 times/day on select days in each wave and assess reflective (e.g., evaluating one’s efficacy, exerting self-control) and reactive processes (e.g., contextual cues) within the dual process framework. We will seek to recruit men and women with a racial/ethnic composition that reflects the demographics in our region. Our overarching hypothesis is that reflective processes will be more strongly linked to behavior among adopters, whereas reactive processes will be more strongly linked to behavior among maintainers. Specific Aim 1 will determine the extent to which momentary reflective and reactive processes are differentially associated with subsequent PA and SB among behavioral adopters and maintainers. Specific Aim 2 will determine the extent to which person-level patterns in reflective and reactive processes predict behavioral adoption versus maintenance at each wave and across the entire year. Specific Aim 3 will explore reflective and reactive motivational processes predicting change in adopter/maintainer status from wave to wave. By determining the reflective and reactive motivational processes and person-level patterns in these processes that positively impact PA and SB, we will improve our understanding of the underlying processes that drive health behaviors in real-time. In turn, this will inform future delivery of personalized intervention content under conditions when the content will be most effective to promote sustained behavior change among older adults. Importantly, this R15 will provide training for 2 undergraduate and 2 graduate students; they will be substantively involved in many aspects of the study, such as participant recruitment, screening, and training, monitoring of compliance, equipment initialization and downloading, data cleaning and analysis, and preparing results for publications and presentations.

PUBLIC HEALTH RELEVANCE: This project seeks to predict and model the adoption and maintenance of optimal levels of physical activity and sedentary behavior among older adults (the fastest growing, least active, and most sedentary segment of the population). The proposed research is

an essential first step towards a larger program of research aimed at integrating real-time data from smartphone-based questionnaires and activity monitors to deliver personalized intervention content when it will be most effective, to promote sustained behavior change among older adults.

CRITIQUE 1

Significance: 2
Investigator(s): 2
Innovation: 2
Approach: 3
Environment: 2

Overall Impact: The proposed prospective cohort study seeks to determine the microtemporal processes (unfolding over minutes, hours, and days) that drive adoption and maintenance of physical activity (PA) and sedentary behavior (SB) among older adults. The investigators propose to recruit two groups – 100 PA adopters and 100 PA maintainers. Participants will complete 3 waves of data (baseline, 6 months, 12 months). Each wave will consist of 14 days of wrist actigraphy monitoring to assess PA and SB and 4 days of EMA (10x/day) to assess reflective (e.g., evaluating one's self-efficacy and exerting self-control) and reactive (e.g., contextual cues) microtemporal processes. The central hypothesis is that reflective processes will be more strongly linked to PA adoption, whereas reactive processes will be more strongly linked to PA maintenance. The significance of the proposed research is high. If successful, the proposed project (which is well supported by rigorous prior research) would advance knowledge of the microtemporal processes in daily life that predict PA and SB – knowledge that could inform the development of future just-in-time interventions designed to promote long-term change in PA and SB. The proposed project is highly likely to generate multiple peer-reviewed publications, will strengthen the research environment, and will expose two undergraduate students to an intensive research experience. The innovation of the proposed research is also high due to its focus on within-person, time-varying microtemporal processes (both reflective and reactive processes) as determinants of both PA adoption and maintenance, which is a potentially important departure from contemporary health behavior theories. Regarding approach, the strengths outweigh the weaknesses. Approach strengths include preliminary data that supports the feasibility of the approach, appropriate frequency and duration of EMA, a carefully described determination PA adoption and maintenance status, valid assessments of key variables, a well-conceived and carefully described analytic plan for each aim, and a thoughtful analysis of potential problems and alternative solutions. In addition, two undergraduate students will be extensively involved in appropriate research tasks and appropriate plans are in place to recruit students belonging to underrepresented groups. Approach weaknesses include potential difficulties in recruiting 100 PA maintainers, issues with obtaining usable accelerometry data during swimming, and a lack of justification for the short window for PA and SB assessment after each EMA prompt (which could exclude relevant data). The investigators are strong – the PI has impressive publication, funding, and undergraduate research mentoring records, and the team possesses the needed expertise and has a history of collaboration. The environment is also strong, providing the needed research resources, community recruitment sites, and pool of well-qualified undergraduate students. As the many strengths of this application far outweighed the identified weaknesses, the overall impact of this application is considered high.

1. Significance:

Strengths

- The application addresses the significant and prevalent problem of poor sustainment of healthy changes in PA and SB in older adults. Existing interventions often fail to have lasting effects on PA and SB.

- If successful, the proposed project would advance knowledge of the microtemporal processes (internal and contextual) in daily life that predict PA and SB. In turn, this knowledge could inform the development of just-in-time interventions to deliver the most appropriate content at the most appropriate time, which ultimately could promote long-term change in PA and SB.
- Prior research that serves as key support appears to be extensive and rigorous, including key evidence from this team and others suggesting that momentary reflective and reactive processes vary within people and that these processes and their variability (instability) are associated with subsequent activity-related behaviors. The rationale for the proposed study is strongly supported by contemporary theories and the extant evidence.
- The proposed project is highly likely to generate multiple peer-reviewed publications making important contributions to the field, will strengthen the research environment at UNC-Greensboro, and will expose two undergraduate students to an intensive research experience.

Weaknesses

- The focus on the two selected groups is appropriate, given the limited scope for the R15 mechanism; however, adding a third group (inactive) would considerably increase the knowledge to be gained (minor).

2. Investigator(s):

Strengths

- PI Maher is an Assistant Professor of Kinesiology with expertise in motivation, PA, SB, EMA, accelerometry, and intensive longitudinal data. She has very strong publication and funding records for her career stage and has a track record of successfully engaging and mentoring undergraduate students in research (including a high percentage of students from underrepresented groups).
- The team of co-investigators and consultants provides complementary expertise in community-based recruitment of older adults, motivation for PA and SB, EMA research, and advanced statistical methods for clustered, intensive longitudinal data and has a history of collaboration.

Weaknesses

- None

3. Innovation:

Strengths

- The focus on within-person, time-varying microtemporal processes as determinants of both PA adoption and maintenance is a potentially important departure from contemporary health behavior theories, which tend to emphasize between-person, time-invariant factors and behavioral adoption only.
- The examination of both reflective and reactive processes is also novel in this context and has the potential to provide new insights into determinants of both PA adoption and maintenance.

Weaknesses

- None

4. Approach:

Strengths

- Preliminary studies support the feasibility of the proposed EMA and actigraphy approach in the target population and this team's ability to recruit and retain participants in multi-wave EMA studies.

- The frequency of EMA (10x random times/day) and duration (three 4-day periods) seems appropriate, balancing data capture and participant burden. EMA items were taken directly or modified from established measures.
- The determination of adoption and maintenance status is carefully described and appropriate, with maintainer status being confirmed by accelerometry data.
- Conducting the introductory sessions at UNCG campus, a community location, or the participants' homes should enhance recruitment.
- Other approach strengths include appropriate eligibility criteria, a carefully described data collection plan, valid assessments of key variables (including the use of separate activity monitors to measure PA and SB), a well-conceived and carefully described analytic plan for each aim, and a thoughtful analysis of potential problems and alternative solutions.
- Over three years (including summers), two undergraduate students will be extensively involved in the proposed project through the execution of tasks appropriate for their level, including recruitment, data collection, and preliminary data management/analysis. These undergraduate students will also have the opportunity to pursue their own independent projects using the collected data under the mentorship of the PI and to present their findings at local and/or national research conferences.
- Appropriate plans are in place to recruit students belonging to underrepresented groups.
- Such an intense and closely mentored research experience is likely to enhanced these students interest in a career in biomedical sciences.

Weaknesses

- Given the low levels of PA in the target population (older adults), recruiting 100 PA maintainers may prove difficult, raising a concern about the feasibility of recruitment for this group. Complicating this issue is the likely possibility that some participants initially classified as maintainers will be reclassified as adopters after examination of the accelerometry data. That said, the planned recruitment efforts at community fitness centers (and letters of support) somewhat allay this concern.
- Given that older adults are likely to engage in low-impact MVPA like swimming (15% in one of their preliminary studies), it seems key for the proposed project to be able to capture PA during that activity. However, as the investigators point out, the Actigraph GT3x-BT is not waterproof and must be removed prior to swimming. The investigators state they will attempt to impute PA data during swimming from the ActivPAL3 (which is waterproof), but the feasibility and validity of such an approach is not clear in the application.
- It is not clear why PA and SB will be operationalized as MVPA during only the 30 minutes after the EMA prompt. It seems that such an approach would exclude meaningful PA and SB data, such as that during 30-60 minutes after an EMA prompt.

5. Environment:

Strengths

- UNC-Greensboro provides the needed research resources for the proposed project, and the partnerships with community fitness centers should facilitate recruitment.
- A pool of well-qualified undergraduate students (who will likely pursue careers in the biomedical sciences) is available at UNC-Greensboro.

Weaknesses

- None

Study Timeline:

Protections for Human Subjects:

Acceptable Risks and/or Adequate Protections

- Risks, protections against risks, potential benefits, and importance of knowledge are all adequately addressed.

Data and Safety Monitoring Plan (Applicable for Clinical Trials Only):

Not Applicable (No Clinical Trials)

Inclusion Plans:

- Sex/Gender: Distribution justified scientifically
- Race/Ethnicity: Distribution not justified scientifically
- For NIH-Defined Phase III trials, Plans for valid design and analysis:
- Inclusion/Exclusion Based on Age: Distribution justified scientifically

Vertebrate Animals:

Not Applicable (No Vertebrate Animals)

Biohazards:

Not Applicable (No Biohazards)

Resource Sharing Plans:

Not Applicable (No Relevant Resources)

Budget and Period of Support:

Recommended budget modifications or possible overlap identified:

- Budget for all three years combined is provided, not each year separately.

CRITIQUE 2

Significance: 3
Investigator(s): 3
Innovation: 4
Approach: 6
Environment: 2

Overall Impact: This R15 proposal examines predictors of physical activity patterns among older adults, with a specific focus on adopting and sustaining physical activity. The proposal uses a rich array of EMA and actigraphy assessments to delineate these associations. Overall the application examines an interesting and important topic, as well as providing a unique training opportunity for students and a strong environment. One approach-related concern is a lack of cohesion between the rich, multidomain data used to characterize predictors of behavior and the outcome metrics, which appear to rely on categorization of sustained activity patterns. With that relatively minor concern, overall enthusiasm for the application is strong.

1. Significance:

Strengths

- Older adults are soon to be over-represented in the U.S. demographic make-up and reducing chronic disease risk through increased physical activity is an important goal
- Understanding predictors of sustained PA at a more granular level could provide important insight to guide future intervention efforts
- The examination of both reflective and reactive motivational processes and behavioral patterns is important and conceptually relevant
- Provides important and clear integration for scientific opportunities among trainees

Weaknesses

- Although the overall study methodology and EMA focus is interesting, the potential knowledge gained does not seem to build substantially from the existing knowledge base examining individual differences in affective change following acute exercise (which may parallel some of the reactive conceptual processes).
- The use of categorization for physical activity metrics is problematic in that this artificial categorization, while clinically meaningful, may inadvertently obscure interesting patterns in the data. This use of categorical analysis also seems to contradict the other strengths of the proposal, which are the granular collection of EMA data. In essence, whereas the explanatory data are captured in a fine-grained, microtemporal fashion, they are then mapped onto summary, artificial categories as the primary outcomes of interest.

2. Investigator(s):

Strengths

- The PI has been quite productive and is focused in this area of research
- The team is well-balanced in terms of conceptual expertise, community outreach connections, and analytical skill sets. All of which increase confidence in the feasibility of the proposal
- It is a particular strength to have expertise in longitudinal modeling approaches, given the focus of the proposal

Weaknesses

- None noted

3. Innovation:

Strengths

- The use of EMA is potentially innovative and informative
- The collection of both reflective and reactive predictors of PA is novel
- The longitudinal examination of PA, with both initiation and sustained components, has not been adequately delineated in prior studies

Weaknesses

- It is unclear whether the present proposal would provide novel inferences above and beyond trait level factors already known to associate with some of the proposed PA outcomes. While the use of microtemporal data collection is potentially interesting, the rationale for this methodology to substantially inform the literature on this topic seems weak as currently presented.
- Given that many individuals may initiate PA only to stop soon after and then initiate again, there seem to be other potential lost opportunities to uncover interesting predictors of PA behavior given the proposed assessment schedule.

4. Approach:

Strengths

- The use of EMA, actigraphy, and other ecological assessments is a strength
- The examination of dual-processes for PA outcomes at different time points of importance (initiation and maintenance) is interesting and a logical extension in this area of study
- The use of microtemporal sampling for EMA data is potentially interesting and informative, although not adequately linked to the core elements of the proposal

Weaknesses

- The complex nature of the longitudinal data collected seems poorly integrated with the outcome metrics, which seem overly simplified given the rich predictor data being sampled.
- Implicit in the classification of individuals as adapter and/or maintainer is the degree of variability among individuals and whether these classifications are truly represented in the population at large. Clearly some individuals will maintain regular exercise and a subset of those will meet conventional guidelines thresholds (e.g. 150 mins), but the stringent use of such a cut-point may prove problematic without a better idea of whether these are true latent classes or putative empirical classes based on using a clinical cut point.

5. Environment:

Strengths

- Strong environment to carry out the proposed study

Weaknesses

- None noted by reviewer

Study Timeline:

Protections for Human Subjects:

Data and Safety Monitoring Plan (Applicable for Clinical Trials Only):

Inclusion Plans:

- Sex/Gender: Distribution justified scientifically
- Race/Ethnicity: Distribution not justified scientifically
- For NIH-Defined Phase III trials, Plans for valid design and analysis:
- Inclusion/Exclusion Based on Age: Distribution justified scientifically

Vertebrate Animals:

Biohazards:

Resource Sharing Plans:

Budget and Period of Support:

Recommend as Requested:

CRITIQUE 3

Significance: 1
Investigator(s): 2
Innovation: 3
Approach: 4
Environment: 2

Overall Impact: This project aims to understand motivational cues for physical activity and sedentary behavior (such as affective states) which potentially rapidly change over time. They will use smartphone-based EMA in older adults intended to adopt physical activity, assessing various motivational processes (reflective, reactive). The goal is to generate foundational knowledge that would then provide the basis for just-in-time adaptive interventions to improve physical activity and sedentary behavior as captured by sensors. The work is novel and well thought-out and considered impactful. It is supported by strong preliminary data demonstrating feasibility and proof of concept. The PI is very experienced in this mode of data collection and analysis. Two concerns arise: the data will be correlational and therefore findings will be exploratory; and, there is perhaps a missed opportunity to use digital phenotyping. Given the more preliminary nature of this project (it is an R15, not an R01) these concerns are felt to be minor and outweighed by the many strengths of the proposal. Beyond the scientific merits, this study is exemplary as an opportunity for students to gain research experience.

1. Significance:

Strengths

- Increasing physical activity and reducing sedentary behavior is very important. It may be the number one public health goal in the US.
- The study has the potential to generate fundamental, widely-applicable insights about motivation.

Weaknesses

- Correlational nature of data makes this less impactful.

2. Investigator(s):

Strengths

- PI has a very strong background and track record.
- Investigative team has all of the requisite expertise for this study.

Weaknesses

- None noted by reviewer

3. Innovation:

Strengths

- Highly innovative conceptually.

- Although EMA cannot still be considered very innovative, this is an innovative use of the technique.

Weaknesses

- Perhaps a missed opportunity to look beyond self-reportable microtemporal processes and use digital phenotyping, and/or gather qualitative feedback.

4. Approach:

Strengths

- Although an observation-only design has some weaknesses, one strength is the large sample size and feasibility of this approach: it should generate strong data in support of the long-term goal of creating just in time adaptive interventions.
- Combining EMA and sensor monitoring to study microtemporal motivational processes is a methodologically strong approach.
- Good attention to both gender and racial/ethnic diversity.

Weaknesses

- None noted by reviewer

5. Environment:

Strengths

- No concerns.

Weaknesses

- None noted by reviewer

Study Timeline:

Protections for Human Subjects:

Acceptable Risks and/or Adequate Protections

- low risk study

Data and Safety Monitoring Plan (Applicable for Clinical Trials Only):

Inclusion Plans:

- Sex/Gender: Distribution justified scientifically
- Race/Ethnicity: Distribution not justified scientifically
- For NIH-Defined Phase III trials, Plans for valid design and analysis:
- Inclusion/Exclusion Based on Age: Distribution justified scientifically

Vertebrate Animals:

Biohazards:

Resource Sharing Plans:

Budget and Period of Support:

Recommend as Requested

THE FOLLOWING SECTIONS WERE PREPARED BY THE SCIENTIFIC REVIEW OFFICER TO SUMMARIZE THE OUTCOME OF DISCUSSIONS OF THE REVIEW COMMITTEE, OR REVIEWERS' WRITTEN CRITIQUES, ON THE FOLLOWING ISSUES:

PROTECTION OF HUMAN SUBJECTS: ACCEPTABLE

INCLUSION OF WOMEN PLAN: ACCEPTABLE

INCLUSION OF MINORITIES PLAN: ACCEPTABLE

INCLUSION ACROSS THE LIFESPAN PLAN: ACCEPTABLE

COMMITTEE BUDGET RECOMMENDATIONS:

Budget for all three years combined is provided, not each year separately.

Presently this looks like a one-year budget that needs to be corrected.

Footnotes for 1 R15 AG066950-01; PI Name: Maher, Jaclyn Parente

+ Derived from the range of percentile values calculated for the study section that reviewed this application.

NIH has modified its policy regarding the receipt of resubmissions (amended applications). See Guide Notice NOT-OD-14-074 at http://grants.nih.gov/grants/guide/notice-files/NOT-OD-14-074.html. The impact/priority score is calculated after discussion of an application by averaging the overall scores (1-9) given by all voting reviewers on the committee and multiplying by 10. The criterion scores are submitted prior to the meeting by the individual reviewers assigned to an application, and are not discussed specifically at the review meeting or calculated into the overall impact score. Some applications also receive a percentile ranking. For details on the review process, see http://grants.nih.gov/grants/peer_review_process.htm#scoring.

MEETING ROSTER

Behavioral Medicine, Interventions and Outcomes Study Section
Risk, Prevention and Health Behavior Integrated Review Group
CENTER FOR SCIENTIFIC REVIEW
BMIO
10/03/2019 - 10/04/2019

Notice of NIH Policy to All Applicants: Meeting rosters are provided for information purposes only. Applicant investigators and institutional officials must not communicate directly with study section members about an application before or after the review. Failure to observe this policy will create a serious breach of integrity in the peer review process, and may lead to actions outlined in NOT-OD-14-073 at https://grants.nih.gov/grants/guide/notice-files/NOT-OD-14-073.html and NOT-OD-15-106 at https://grants.nih.gov/grants/guide/notice-files/NOT-OD-15-106.html, including removal of the application from immediate review.

CHAIRPERSON(S)

FILLINGIM, ROGER B, PHD
DISTINGUISHED PROFESSOR
DEPARTMENT OF COMMUNITY DENTISTRY
AND BEHAVIORAL SCIENCE
COLLEGE OF DENTISTRY
UNIVERSITY OF FLORIDA
GAINESVILLE, FL 32610

MEMBERS

BARRETT, A M, MD
PROFESSOR
DEPARTMENT OF PHYSICAL MEDICINE
AND REHABILITATION
RUTGERS NEW JERSEY MEDICAL SCHOOL
WEST ORANGE , NJ 07052

BRUEHL, STEPHEN, PHD
PROFESSOR
DEPARTMENT OF ANESTHESIOLOGY
SCHOOL OF MEDICINE
VANDERBILT UNIVERSITY MEDICAL CENTER
NASHVILLE, TN 37212

BURG, MATTHEW M, PHD
PROFESSOR
DEPARTMENT OF INTERNAL MEDICINE
YALE UNIVERSITY SCHOOL OF MEDICINE
NEW HAVEN, CT 06520

BURNS, JOHN W., PHD *
PROFESSOR
DEPARTMENT OF BEHAVIORAL SCIENCES
RUSH UNIVERSITY MEDICAL CENTER
CHICAGO, IL 60612

CHARVET, LEIGH, PHD *
ASSOCIATE PROFESSOR
NEW YORK UNIVERSITY MEDICAL CENTER
NEW YORK, NY 10016

COHEN, RONALD A, PHD *
PROFESSOR AND DIRECTOR
CENTER FOR COGNITIVE AGING AND MEMORY
MCKNIGHT BRAIN INSTITUTE
UNIVERSITY OF FLORIDA
GAINESVILLE, FL 32610

DITRE, JOSEPH W, PHD
ASSOCIATE PROFESSOR
DEPARTMENT OF PSYCHOLOGY
SYRACUSE UNIVERSITY
SYRACUSE, NY 13104

DURANT, RAEGAN W, MD, MPH
ASSOCIATE PROFESSOR
DEPARTMENT OF MEDICINE
DIVISION OF PREVENTIVE MEDICINE
UNIVERSITY OF ALABAMA AT BIRMINGHAM
BIRMINGHAM, AL 35294

ESTABROOKS, PAUL, PHD *
PROFESSOR
DEPARTMENT OF HEALTH PROMOTIONS,
SOCIAL & BEHAVIORAL HEALTH
COLLEGE OF PUBLIC HEALTH
UNIVERSITY OF NEBRASKA MEDICAL CENTER
OMAHA, NE 68198

HASSETT, AFTON L, PSYD *
ASSOCIATE RESEARCH SCIENTIST
DEPARTMENT OF ANESTHESIOLOGY
CHRONIC PAIN AND FATIGUE RESEARCH CENTER
UNIVERSITY OF MICHIGAN MEDICAL SCHOOL
ANN ARBOR, MI 48106

HOULE, TIMOTHY T, PHD *
ASSOCIATE PROFESSOR
ANESTHESIA-MASSACHUSETTS GENERAL HOSPITAL
HARVARD MEDICAL SCHOOL
BOSTON, MA 02114

HUI, DAVID, MD
ASSOCIATE PROFESSOR
DEPARTMENT OF PALLIATIVE CARE AND
REHABILITATION MEDICINE
UNIVERSITY OF TEXAS MD ANDERSON CANCER CENTER
HOUSTON, TX 77030

JENSEN, MARK P, PHD
PROFESSOR AND VICE CHAIR FOR RESEARCH
DEPARTMENT OF REHABILITATION MEDICINE
UNIVERSITY OF WASHINGTON
SEATTLE, WA 98195

KNIGHT, SARA J, PHD
PROFESSOR AND RESEARCH SCIENTIST
DIVISION OF EPIDEMIOLOGY
DEPARTMENT OF INTERNAL MEDICINE
UNIVERSITY OF UTAH
SALT LAKE CITY, UT 84132

LENGACHER, CECILE A, PHD *
PROFESSOR AND DIRECTOR
BS-PHD PROGRAM
COLLEGE OF NURSING
UNIVERSITY OF SOUTH FLORIDA
TAMPA, FL 33612-4766

LENZE, ERIC J, MD
PROFESSOR
DEPARTMENT OF PSYCHIATRY
WASHINGTON UNIVERSITY SCHOOL OF MEDICINE
SAINT LOUIS, MO 63110

LUYSTER, FAITH S, PHD *
ASSISTANT PROFESSOR
SCHOOL OF NURSING
UNIVERSITY OF PITTSBURGH
PITTSBURGH, PA 15261

MA, JUN, MD, PHD *
BETH AND GEORGE VITOUX PROFESSOR OF MEDICINE
DEPARTMENT OF MEDICINE
COLLEGE OF MEDICINE
UNIVERSITY OF ILLINOIS AT CHICAGO
CHICAGO, IL 60608

MATHEW, JOSEPH P, MD, MHSC
PROFESSOR
DEPARTMENT OF ANESTHESIOLOGY
DUKE UNIVERSITY MEDICAL CENTER
DURHAM, NC 27710

PALESH, OXANA G, PHD
ASSOCIATE PROFESSOR
DEPARTMENT OF PSYCHIATRY AND BEHAVIORAL
SCIENCES
STANFORD CANCER INSTITUTE
STANFORD UNIVERSITY
STANFORD, CA 94305

PATTON, SUSANA R, PHD
CENTER DIRECTOR
CENTER FOR HEALTHCARE DELIVERY SCIENCE
NEMOURS CHILDRENS HEALTH SYSTEM
JACKSONVILLE, FL 32259

PERRY, TAMARA T, MD
ASSOCIATE PROFESSOR
DEPARTMENT OF PEDIATRICS
DIVISION OF ALLERGY IMMUNOLOGY
UNIVERSTIY OF ARKANSAS FOR MEDICAL SCIENCES
LITTLE ROCK, AR 72202

PEUGH, JAMES, PHD *
RESEARCH ASSOCIATE PROFESSOR
DEPARTMENT OF PEDIATRICS
DIVISIONS OF BEHAVIORAL MEDICINE AND CLINICAL
PSYCHOLOGY AND BIOSTATISTICS AND EPIDEMIOLOGY
CINCINNATI CHILDREN'S HOSPITAL AND MEDICAL CENTER
CINCINNATI, OH 45229--302

RINI, CHRISTINE, PHD
PROFESSOR
DEPARTMENT OF MEDICAL SOCIAL SCIENCES
NORTHWESTERN UNIVERSITY FEINBERG
SCHOOL OF MEDICINE
CHICAGO, IL 60611

SMITH, PATRICK J, PHD
ASSOCIATE PROFESSOR
DEPARTMENT OF PSYCHIATRY AND BEHAVIORAL MEDICINE
DUKE UNIVERSITY
DURHAM, NC 27710

STEWART, JESSE C, PHD
PROFESSOR
DEPARTMENT OF PSYCHOLOGY
INDIANA UNIVERSITY-PURDUE UNIVERSITY
INDIANAPOLIS
INDIANAPOLIS, IN 46202

UEBELACKER, LISA A, PHD
PROFESSOR
PSYCHOSOCIAL RESEARCH
BUTLER HOSPITAL
BROWN UNIVERSITY
PROVIDENCE, RI 02906

WEN, KUANG-YI, PHD *
ASSOCIATE PROFESSOR
DIVISION OF POPULATION SCIENCE
SIDNEY KIMMEL CANCER CENTER
THOMAS JEFFERSON UNIVERSITY
PHILADELPHIA, PA 19107

SCIENTIFIC REVIEW OFFICER

MANN, LEE S, PHD
SCIENTIFIC REVIEW OFFICER
CENTER FOR SCIENTIFIC REVIEW
NATIONAL INSTITUTES OF HEALTH
BETHESDA, MD 20892

EXTRAMURAL SUPPORT ASSISTANT

WATTS, MELISSA
EXTRAMURAL SUPPORT ASSISTANT
CENTER FOR SCIENTIFIC REVIEW
NATIONAL INSTITUTE FOR HEALTH
BETHESDA, MD 20892

* Temporary Member. For grant applications, temporary members may participate in the entire meeting or may review only selected applications as needed.

Consultants are required to absent themselves from the room during the review of any application if their presence would constitute or appear to constitute a conflict of interest.
